# Intergenerational Communication as Intervention: Social Isolation in Older Adults During COVID-19

**DOI:** 10.1093/geroni/igab046.296

**Published:** 2021-12-17

**Authors:** Laura Kirk, Penny Kessler, Stephanie Gingerich, Sharon McGill, Hanna Pryor

**Affiliations:** 1 University of Minnesota, Minneapolis, Minnesota, United States; 2 University of Minnesota, University of Minnesota School of Nursing, Minneapolis, Minnesota, United States; 3 University of Minnesota School of Nursing, Minneapolis, Minnesota, United States; 4 Presbyterian Homes and Services, Roseville, Minnesota, United States

## Abstract

Social isolation and loneliness are prevalent and impactful in the lives of older adult across care settings, and the emergence of a deadly global pandemic requiring social distancing and quarantining exacerbated these experiences significantly in 2020. A semester-long communication-focused clinical project was developed and piloted for sophomore bachelor of nursing science (BSN) students during fall 2020. Affording preclinical nursing students the opportunity to develop communication skills early in their program of study holds potential, and learning the story of older adults appears to be mutually beneficial; older adults serve as mentors and share their story, and preclinical nursing students have an opportunity to learn about their mentor’s life, challenging some prevalent stereotypes about aging. A pre- post-clinical survey of student attitudes toward older adults suggests a dramatic positive shift in perspective, and unsolicited, anecdotal comments in student reflections support this finding: “...it helped me feel much more open to working with older adults in the future”; “This conversation taught me that my assumptions about the older generation are not always correct”. Of older adult participants, 96% reported the experience enriched and enhanced their social connectedness, with 88% requesting to participate in the program again. Narrative comments from residents indicated that their involvement enabled them to feel engaged and purposeful: “I was a mentor”; “Conversations were so alive. Connections with curious young people fill my heart and soul.” Intergenerational sharing of life stories has the potential for both healing and growth and may provide an antidote to ageism.

